# Influence of metformin intake on the risk of bladder cancer in type 2 diabetes patients

**DOI:** 10.1186/2049-3258-73-S1-P3

**Published:** 2015-09-17

**Authors:** Maria (Mieke) Goossens, Frank Buntinx, Maurice Zeegers, Annemariek Driessen, Marie De Bruin, Frank De Vries

**Affiliations:** 1 KU Leuven, Leuven, Belgium; 2 University Maastricht, Maastricht, Netherlands; 3 Utrecht University, Utrecht, Nederland

## Objective

The aim of this study is to look at the influence of metformin intake and duration, on urinary bladder cancer (UBC) risk, with sulfonylurea (SU) only users as control using a new-user design (inception cohort).

## Methods

We conducted a retrospective cohort study using data from the UK Clinical Practice Research Datalink (CPRD) including all patients with at least one prescription of oral anti-diabetic drugs (ADD) and/or insulin and aged 18 years or older. The risk of UBC in different groups of ADD users (metformin alone (1), metformin in combination (2) with other ADD medication (glinides, glitazones, DPP-4-inhibitors, SUs, insulin or more than one combination), all metformin users (1 + 2) was compared with SU only users using Cox proportional hazards models. The estimates were adjusted for age, gender, smoking status, BMI and diabetes duration.

## Results

The inception cohort included 165,398 participants of which 132,960 metformin users and 32,438 SU only users. During a mean follow-up time of more than five years 693 patients developed UBC, 124 of the control group and 461 of the all metformin users. There was no association between metformin intake and UBC risk (HR = 1.12 (95% CI 0.90-1.40)) compared to SU only users, even after adjustment for diabetes duration (HR = 1.13 (95% CI 0.90-1.40)). We found a pattern of decreasing risk of UBC with increasing duration of metformin intake, which was statistically not significant.

## Conclusion

Metformin has no influence on the risk of UBC compared to SU in type 2 diabetes patients using a new-user design.

